# Detection and Severity Classification of Sleep Apnea Using Continuous Wearable SpO_2_ Signals: A Multi-Scale Feature Approach

**DOI:** 10.3390/s25061698

**Published:** 2025-03-09

**Authors:** Nhung H. Hoang, Zilu Liang

**Affiliations:** 1 Ubiquitous and Personal Computing Lab, Kyoto University of Advanced Science (KUAS), Kyoto 615-8577, Japan; 2023md05@kuas.ac.jp; 2 Institute of Industrial Science, The University of Tokyo, Tokyo 153-8505, Japan

**Keywords:** sleep apnea, machine learning, feature engineering, SpO_2_, wearable

## Abstract

The use of wearable devices for sleep apnea detection is growing, but their limited signal resolution poses challenges for accurate diagnosis. This study explores the feasibility of using SpO_2_ signals from wearable sensors for detecting sleep apnea and classifying its severity. We propose a novel multi-scale feature engineering approach, which extracts features from coarsely grained SpO_2_ signals across timescales ranging from 1 s to 600 s. Our results show that traditional SpO_2_ markers, such as the oxygen desaturation index (ODI) and Lempel–Zip complexity, lose their relevance with the Apnea–Hypopnea Index (AHI) at longer timescales. In contrast, non-linear features like complex entropy, sample entropy, and fuzzy entropy maintain strong correlations with AHI, even at the coarsest timescales (up to 600 s), making them well suited for low-resolution data. Multi-scale feature extraction improves model performance across various machine learning algorithms by alleviating model bias, particularly with the Bayes and CatBoost models. These findings highlight the potential of multi-scale feature engineering for wearable device applications where only low-resolution data are commonly available. This could improve accessibility to low-cost, at-home sleep apnea screening, reducing reliance on expensive and labor-intensive polysomnography. Moreover, it would allow even healthy individuals to proactively monitor their sleep health at home, facilitating the early identification of potential sleep problems.

## 1. Introduction

In recent decades, the self-monitoring of health information has become a prominent trend, with widespread applications in healthcare [1]. Advancements in compact, sensor-integrated devices now enable the collection of various health metrics without requiring visits to medical facilities [2]. For sleep health, such devices—designed to be non-invasive and portable—offer a means to monitor sleep patterns consistently over extended periods, providing valuable insights into daily and longitudinal changes with reasonable validity [3,4]. These tools highlight a shift toward accessible and continuous health tracking, improving convenience and patient autonomy in managing well-being [5].

Despite the increasing adoption of wearable devices for health monitoring, their application in sleep studies remains limited. These devices have not yet received FDA approval as replacements for polysomnography (PSG), the gold standard for diagnosing sleep-related disorders. Furthermore, their limitations include reduced accuracy, reliability, comprehensiveness, and data granularity compared to PSG [6]. Compact and convenient designs often come at the cost of information quality, as they rely on a limited number of sensors—such as photoplethysmography (PPG), accelerometers, and heart rate monitors—while excluding comprehensive measurements like electroencephalograms (EEG), electrocardiograms (ECG), and respiratory movement data.

Nevertheless, wearable devices offer undeniable benefits. First, wearable devices enable users to move more freely during sleep, as they minimize the need for physical connections between sensors and recording devices. This design supports a more natural and comfortable sleeping experience, reducing potential disruptions caused by traditional polysomnography setups. Additionally, home-based sleep data collection has emerged as a more effective alternative for reflecting sleep patterns in a natural environment [7]. This approach enhances ecological validity by capturing sleep behaviors under real-world conditions and improves ergonomic value by minimizing constraints on user mobility and comfort. Second, wearable devices have the capacity to collect data continuously and over long periods, addressing a critical gap in traditional sleep studies. The “first-night effect”, a well-documented phenomenon in sleep research, persists despite advancements in measurement environments and equipment quality [8]. This issue underscores the limitations of relying on isolated polysomnography sessions. Even with large datasets like the Sleep Heart Health Study (SHHS) and the Wisconsin Sleep Cohort (WSC), longitudinal tracking has been constrained. These datasets show a significant drop in participant follow-ups, with intervals often extending to 5 years or more. Thus, over a span of 5 years, the SHHS dataset provides only about two sleep recordings per individual for reference. The WSC dataset includes more longitudinal measurements but features fewer participants, with only 566 individuals (50.40%) returning for a third measurement and just 121 (10.77%) for a fourth. This limited frequency of recordings underscores significant challenges in addressing the timeliness and urgency of monitoring and diagnosing sleep-related disorders. Furthermore, it highlights gaps in the “gold-standard” approach, particularly regarding its capacity to track disease progression and facilitate subsequent treatment effectively. Wearable devices, offering frequent and continuous data collection, have the potential to bridge this gap [9,10].

Blood oxygen saturation (SpO_2_) has emerged as an important signal for sleep apnea detection, with numerous studies demonstrating its diagnostic value. SpO_2_ has been found to outperform other signals, such as ECG or PPG, in classification tasks related to sleep apnea [11,12,13]. The widespread integration of SpO_2_ sensors in wearable devices makes this signal particularly accessible for continuous, home-based health monitoring, further cementing its potential as a key marker in the development of practical solutions for sleep apnea detection.

There are three primary methodologies for diagnosing sleep apnea: detecting individual apneic events, regressing the Apnea–Hypopnea Index (AHI), and classifying sleep apnea severity. Early research focused on detecting individual apneic events, often breaking down the epoch length from 30s to smaller intervals with the aim of building real-time detection systems [14,15]. However, a limitation of SpO_2_ as a diagnostic signal lies in its delayed response to apneic events. The drop in blood oxygen saturation is not immediate following an apneic event; it typically delays by 10–40 s due to the time required for oxygen levels to decrease [16,17,18]. This delay introduces challenges in accurately capturing the temporal dynamics of apneic events, which is crucial for timely diagnoses and interventions.

While detecting apneic episodes is best suited for high-frequency signals like those from polysomnography, wearable devices often face limitations in terms of signal granularity and resolution. Despite these challenges, wearable devices are more appropriate for severity classification, which focuses on identifying broad patterns rather than precise event timing. Severity classification is particularly advantageous for large datasets or long-term monitoring, where computational demands are lower, and interpretability is simpler. Recent advances in wearable sleep-monitoring technologies, though limited in signal resolution, are well suited for this task [19].

In this study, we investigate the feasibility of detecting sleep apnea and classifying its severity using SpO_2_ signals only, which can be continuously measured with wearable sensors. We proposed a novel feature engineering method that extracts features from coarsely grained signals across multiple timescales, ranging from 1 s to 600 s. This method is compared to a baseline method that extracts features from a single timescale at 1 s, which is commonly used in prior research for its detailed temporal resolution [20,21,22,23,24,25,26]. We thoroughly examine how feature relevance changes as the timescale increases and evaluate the corresponding impact on model performance. In particular, this study aims to answer the following research questions:

- RQ1: How do different timescales impact the relevance of features for sleep apnea severity?
- RQ2: Which features, derived from multiple timescales, are most useful for detecting sleep apnea and classifying its severity?
- RQ3: Which machine learning algorithms perform best with multi-scale feature engineering?

We choose to implement machine learning techniques because they have demonstrated effectiveness in previous sleep apnea studies. More specifically, we intentionally adopt traditional machine learning techniques over deep learning approaches. This decision is driven by the fact that, unlike deep learning, models developed using traditional machine learning techniques are often more interpretable, which makes them better suited for healthcare applications where transparency and explainability are important [27]. By building more interpretable models, we aim to provide evidence in the form of clear, measurable outcomes that demonstrate how features derived from SpO_2_ signals across varied time resolutions relate to sleep apnea severity. This evidence will help highlight the clinical relevance of these features, showing how they correlate with meaningful healthcare outcomes. We hope that by providing these insights, this study will enhance the transparency of AI-based sleep apnea screening models and ultimately improve their adoption in healthcare settings.

## 2. Materials and Methods

### 2.1. Dataset

In this study, we utilize the Sleep Heart Health Study (SHHS) dataset [28,29], one of the largest public sleep datasets. To minimize data interdependency, we focus on the data collected during the first visit of each subject, which includes 5,787 sleep recordings with a balanced gender distribution. Following preprocessing and feature extraction, we exclude poor-quality features and suboptimal sleep recordings, resulting in a final dataset of 4,664 sleep recordings for the subsequent analysis. The demographic information for the subjects used for the model development is summarized in Table 1. For denoising the SpO_2_ signals, we remove SpO_2_ readings below 60% or above 100%, as these values are considered physiological outliers or measurement errors [13,30,31,32].

### 2.2. Multi-Scale Features Engineering

**Figure 1:**
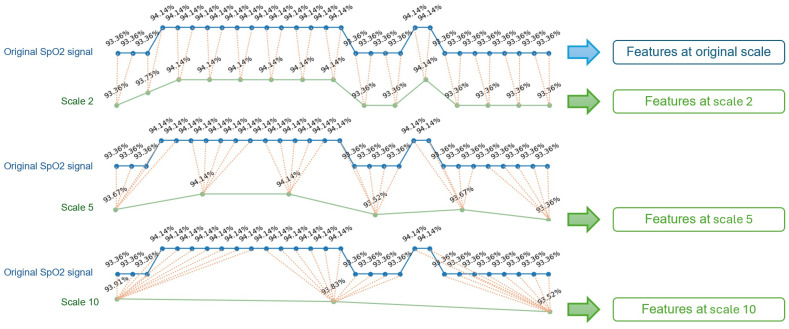
Illustration for coarse-graining procedure, for timescales of 2, 5, and 10 s.

The original SpO_2_ signals in the SHHS dataset are sampled at 1 Hz. The multi-scale feature engineering process, inspired by existing studies  [24,33], begins with coarse-graining the signals at various timescales, ranging from 2 to 10 s with an increment of 1 s, from 15 to 60 s with an increment of 5 s, and from 120 to 600 s with an increment of 60 s. This allows us to examine the predictive power of features at a wide range of timescales. Multiple coarse-grained timescales are constructed by averaging the data points within non-overlapping windows of increasing length. The coarse-graining process is illustrated in Figure 1.

**Figure 2:**
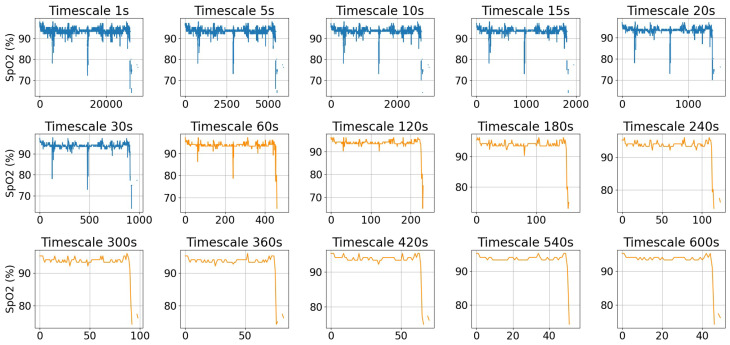
An example of a SpO_2_ signal at its original timescale of 1 s and after coarse-graining at various timescales. The orange plots illustrate the low-resolution data typically available from consumer smartwatches such as Fitbit.

Figure 2 shows an example of a SpO_2_ signal at its original sampling rate and after coarse-graining at various timescales, ranging from 5 s to 600 s. As the timescale increases, the signal becomes smoother, and large fluctuations are filtered out, leading to a reduction in complexity. The orange plots in Figure 2 illustrate the low-resolution data typically accessible from consumer smartwatches such as Fitbit. It is observed that at a timescale of 60 s, the signal is noticeably smoother while still retaining key fluctuations.

For each timescale of the coarse-grained SpO_2_ signal, we extract 106 features, which include time domain, frequency domain, non-linear features, and oximetry biomarkers. The oximetry biomarkers are derived using the open-source POBM toolbox [25], which analyzes oximetry time series data to extract relevant metrics for further analysis. The non-linear features are extracted using Python libraries [34,35]. After extracting the features at each timescale, we combine the features across all timescales. Features with more than 20% missing values or more than 40% zero values are excluded.

To evaluate the impact of timescales on the predictive power of features, we first calculate the Spearman’s correlation coefficient between individual features and the Apnea–Hypopnea Index (AHI). Spearman’s correlation is a non-parametric measure that assesses the strength and direction of a monotonic relationship between two variables [36]. We choose it over Pearson’s correlation due to its robustness to outliers, loose assumptions on data distributions, and suitability for detecting non-linear associations. The Spearman’s correlation coefficient (ρ) ranges from −1 to 1, where −1 and 1 represent the perfect negative and positive monotonic relationship, respectively. To visualize the relationships between features and AHI, we also create scatter plots for each feature-AHI pair. For this study, we specifically use the variable nsrr_ahi_hp3r_aasm15, which adheres to the AASM 2015 scoring rules to ensure standardization and comparability across analyses [37].

### 2.3. Model Training, Validation and Testing

The dataset is split into 80% for training and 20% for testing. To ensure that each feature contributes equally to the learning process of the model, we apply standard scaling, which transforms the features to have a mean of 0 and a standard deviation of 1.

We apply multiple machine learning algorithms including naïve Bayes (NB), Logistic Regression (LR), decision tree (DT), K-nearest neighbor (KNN), CatBoost, XGBoost (XGB), and a basic multilayer perceptron (MLP). These algorithms are chosen for their simplicity and interpretability, which makes them well suited for understanding feature importance. Hyperparameter tuning is achieved through grid search with 5-fold cross-validation. A baseline single-scale model is developed using features extracted from the original 1 Hz SpO_2_ signal. Model performance is evaluated using several metrics, including accuracy (Acc), precision (Pre), sensitivity (Sen), F1-score, Matthews correlation coefficient (MCC), and area under curve (AUC). To gain a statistical perspective of the model performance, the training and test process is repeated 50 times, each time using a different random seed for data splitting. This approach helps minimize the risk that the model performance is influenced by a specific train–test split and confirms that the observed behavioral patterns of multi-scale features are consistent across experiments rather than being due to random variation. The results are reported as the mean and standard deviation in Section 3.

**Figure 3:**
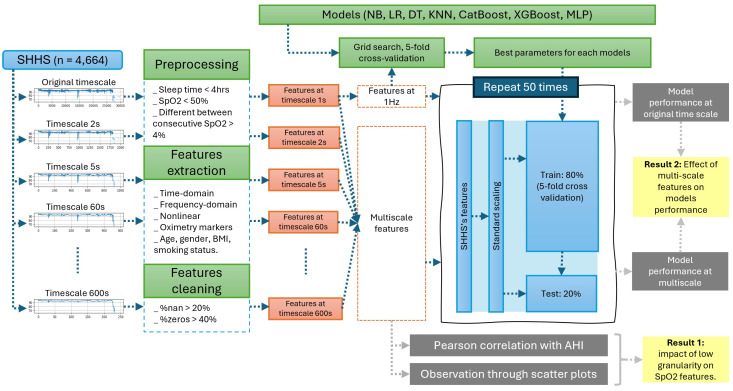
Flowchart of analyses and methods.

Figure 3 provides a visual summary of the research methodology. It begins with the input data and proceeds through feature extraction, followed by hyperparameter selection for optimization, and concludes with the training and testing of the machine learning models.

## 3. Results

### 3.1. Feature Utility Across Different Signal Granularity Scales

**Figure 4:**
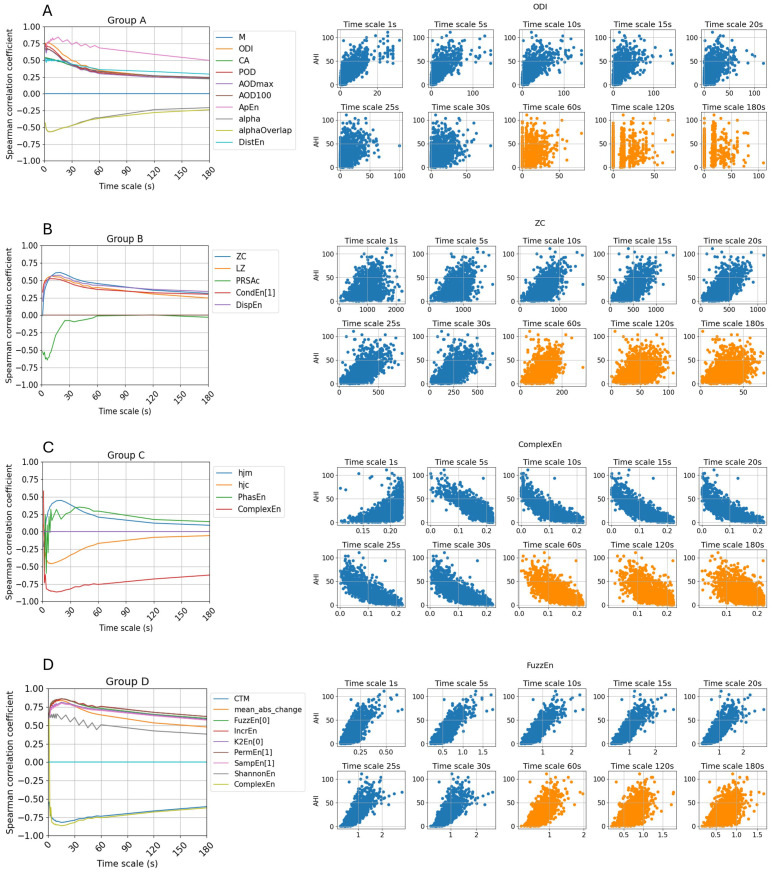
Three interesting trends regarding correlation coefficient between features and AHI at multi-scale are visualized in subplot (**A**–**C**). Subplot (**D**) visualizes the important features that remain high correlation with apnea severity across all timescales.

After analyzing the results of the Spearman correlation coefficient and visualizing the data through scatter plots, several interesting trends regarding the impact of coarse-graining on feature behavior are identified. Figure 4 provides a summary of these four trends. Table 2 summarizes the maximum and minimum correlation values, along with the corresponding timescale at which the highest correlation is observed.

First, as illustrated in Figure 4A, we examine features previously identified as important for sleep apnea detection in both epoch-wise and subject-wise classification. The line plot for group A reveals a notable trend: while the correlation between these features and AHI is initially strong, it declines rapidly as the timescale increases. By the 30 s timescale, the correlation value is reduced to approximately half of its original magnitude. At the 60 s timescale, the correlation fluctuates around 0.25, indicating a weak association. In half of the features in Group A, the highest correlation values are predominantly observed at the 1 s timescale. For the remaining features, the maximum correlation occurs at larger timescales but not longer than 5 s. However, the difference between this peak value and the correlation at 1 s remains relatively small, ranging from 0.03 to 0.08.

In this group, the approximate entropy demonstrates a more stable relationship with AHI compared to other features, with a median correlation of 0.72 and maintaining a correlation above 0.5 even at a timescale of 120 s (equivalent to a sampling frequency of one sample per two minutes). However, as the timescale increases further, its correlation declines to nearly zero. In contrast, the oxygen desaturation index (ODI), despite having the highest correlation at 1 s and being widely used in sleep apnea detection, exhibits a sharp decline as the timescale increases. As observed in the scatter plot in Figure 4A, the feature values become segmented, with a growing proportion of zero values, indicating that desaturation events are harder to detect when the SpO_2_ signals are coarsely grained at longer timescales.

The features in Figure 4B exhibit an opposite trend compared to Group A. Some features that were initially uncorrelated when derived from the original SpO_2_ signals exhibit stronger correlations after the signals are coarse-grained. For instance, zero-crossing (ZC), which in this context represents mean crossing since SpO_2_ values are never lower than 60%, reaches a strong correlation of 0.61 at the 20 s timescale, whereas at 1 s, it shows no correlation with AHI with a correlation coefficient at −0.01. Notably, the PRSAc feature shows a sharp increase in correlation, reaching −0.68 at the 6 s timescale. While PRSAc does not consistently exhibit a strong relationship with AHI across other timescales, its high correlation at 6 s suggests it could serve as a valuable indicator of sleep apnea severity at this specific data resolution.

With the exception of the PRSAc feature, the other features in this group exhibit their highest correlation with AHI at timescales of 10 s or higher. At the 60 s timescale, some features still maintain a relatively strong correlation with AHI, with ZC at 0.45, Lempel–Zip (LZ) complexity at 0.39, and dispersion entropy (DispEn) at 0.43. This indicates that despite the reduced signal resolution, these features retain meaningful associations with sleep apnea severity, highlighting their potential across different granularities.

Furthermore, several features, including Hjorth mobility (HjM), Hjorth complexity (HjC), phase entropy (PhasEn), and complex entropy (ComplexEn), exhibit correlation polarity shifts, transitioning from positive to negative correlations or vice versa. In particular, as can be seen from the scatter plots in Figure 4C, ComplexEn initially shows a positive correlation with AHI at the 1 s timescale but transitions to a negative correlation at larger timescales. Notably, ComplexEn achieves the highest absolute correlation among all surveyed features, reaching −0.86. Furthermore, this feature maintains a moderate correlation even at larger timescales, with values of −0.85 at 10 s, and −0.75 at 60 s, indicating its potential as a robust marker for sleep apnea severity across different data resolutions. Changes in correlation polarity provide valuable insights into the non-linear relationship between features and sleep apnea severity as the data resolution decreases.

**Figure 5:**
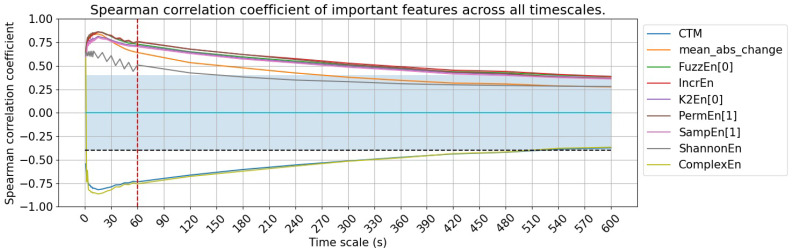
Spearman correlation coefficient of important features. The blue area indicates where the absolute value of the correlation coefficient is lower than 0.4, showing a weak relationship area.

Finally, we identify a set of important features that maintain consistently moderate to strong correlations across all timescales, with correlation coefficients exceeding 0.4 at all timescales below 60 s. These nine features are summarized in the last nine rows of Table 2 and illustrated in Figure 4D, which include two time-domain features and seven non-linear features. Figure 5 provides better visualization of the timescale from 1s to 600s. Their consistent performance across timescales allows for effective sleep apnea detection while optimizing storage and computational efficiency, making them particularly well suited for applications in consumer wearable devices.

### 3.2. Model Performance

To evaluate the effectiveness of the multi-scale approach in model development, we compare models trained on multi-scale features to baseline models trained on single-scale features derived at the original SpO_2_ signals with a resolution of 1 s.

#### 3.2.1. Binary Classification

**Figure 6:**
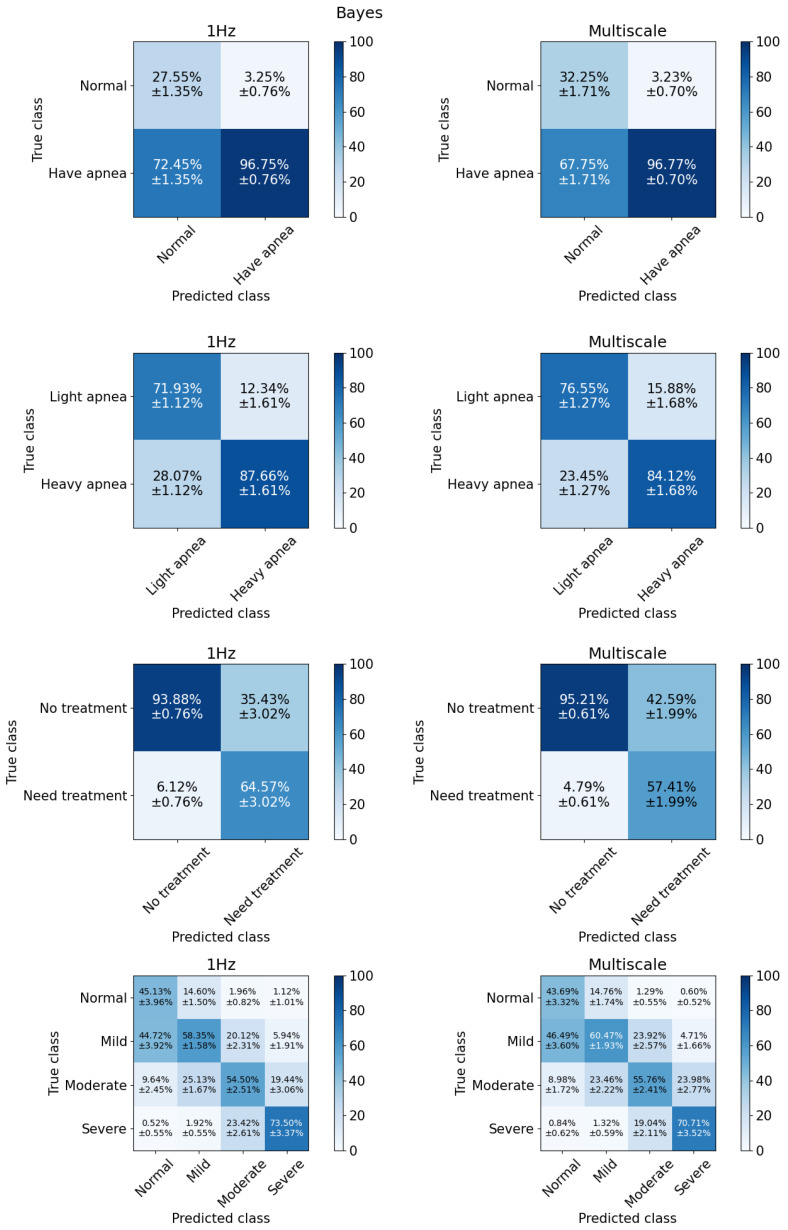
The Bayes model failed to distinguish normal and apnea patients, but the result slightly improved with multi-scale features.

**Figure 7:**
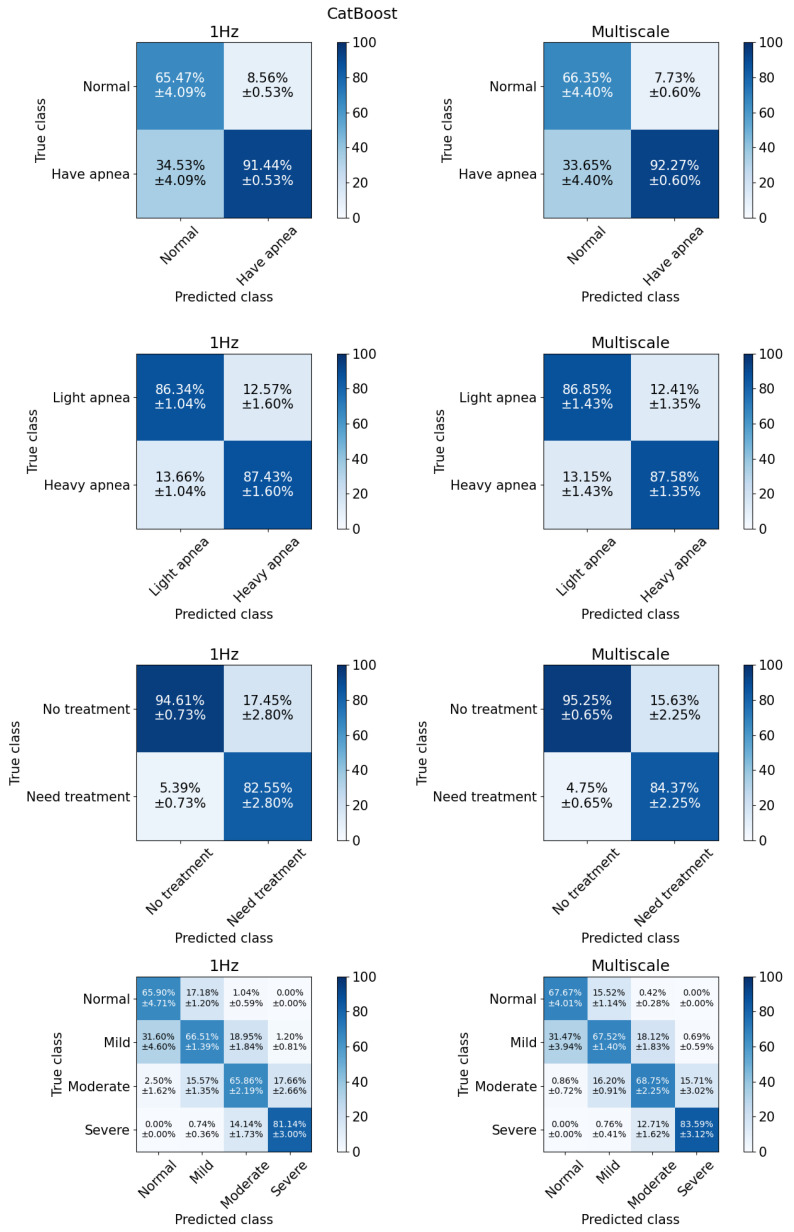
The CatBoost model achieved the highest performance for all tasks in general.

For binary classification, we evaluate model performance using three AHI cut-offs: 5, 15, and 30 events per hour. These cut-offs serve different clinical purposes [38]. At cut-off 5, the classification differentiates between normal and apneic patients. Cut-off 15 represents the distinction between individuals with mild symptoms and those with more severe symptoms. Meanwhile, cut-off 30 serves as a threshold to separate individuals who do not yet require treatment from those who should seek medical intervention or assistance. Figure 6 and Figure 7 show the classification results for the Bayes and CatBoost models, respectively.

For cut-off ≥ 5 events/h, subjects were categorized into two groups: healthy individuals and those with sleep apnea, labeled as “normal” and “have apnea”, respectively. We observed that classifiers at this cut-off often result in the highest degree of bias among the models used in this study. The poorest performance is observed in the first row of Figure 6, where the Bayes model exhibits a strong bias toward the majority class (patients).

The results from training and testing the Bayes model using features extracted from a single timescale of 1 s indicate that the model failed to distinguish between the two groups, classifying most of the subjects in the test set as having sleep apnea. However, incorporating multi-scale features significantly improved performance, increasing the average TN rate to 32.21%. Meanwhile, it is noteworthy that multi-scale features could not completely eliminate the model’s bias toward the majority class as reflected in the significantly lower MCC of the Bayes model in Table 3. On the other hand, with the multi-scale features, the accuracy, sensitivity, MCC increased by 6.42%, 7.68%, 0.06, respectively, while precision remained unchanged. This indicates that the model achieved better class balance in distinguishing between healthy and sleep apnea subjects when provided with the multi-scale feature set.

For cut-off ≥ 15 events/h, the classification task involves distinguishing individuals with mild or no apnea from those with moderate to severe apnea, labeled as “Light apnea” and “Heavy apnea”. The ratio between the two classes is almost the same, with 52.02% for the group of people with light sleep apnea and 47.98% for the group of people with heavy apnea. This is also the case where the models have good results and are balanced between the two classes.

Once again, the Naïve Bayes model shows the most notable improvement with multi-scale features, with accuracy increasing by 2.41%, sensitivity by 9.84%, and MCC by 0.03, while precision is decreased by 3.55%. Despite the reduction in precision, the model exhibits a better balance in classifying both groups. Multi-scale features also enhance the accuracy, precision, and sensitivity of the MLP models by approximately 2–4%.

For cut-off ≥ 30 events/h, the classification task distinguishes individuals requiring treatment from those who do not, with class labels “No treatment” and “Need treatment”, as severe apnea typically necessitates medical intervention or sleep aids. Similar to the AHI ≥ 5 case, the models face challenges due to class imbalance. Classification performance remains relatively strong, with MCC values ranging from 0.61 to 0.74. However, for the Naïve Bayes and Logistic Regression models, the introduction of multi-scale features has an adverse effect. Both models exhibit a trade-off between precision and sensitivity: for Naïve Bayes, sensitivity increases by 7.32% while precision decreases by 7.16%, and for Logistic Regression, sensitivity increases by 10.58% while precision decreases by 9.65%. Consequently, the models tend to misclassify severe apnea cases into the milder apnea category, reducing their ability to accurately identify individuals requiring treatment.

The observed increase in MCC, particularly at the AHI cut-off of 5, suggests that multi-scale feature engineering improves class separation. Specifically, as shown in the first row of confusion matrix in Figure 6, this approach increases the percentage of true positives while reducing false positives. In the case of CatBoost as shown in Figure 7, false negatives are also slightly decreased.

#### 3.2.2. Multiclass Classification

In the multiclass classification scenario, the models attempt to classify four levels of sleep apnea severity, normal, mild, moderate, and severe, with class distributions of 13.59%, 38.42%, 30.32%, and 17.67%, respectively. Misclassification remains a challenge, particularly between the normal and mild apnea groups, which exhibit the highest error rates. As shown in the last section of Table 3, CatBoost achieves the best performance, with an accuracy of 70.46%, precision of 71.88%, sensitivity of 67.64%, and an MCC of 0.58. XGBoost and MLP follow closely behind, but CatBoost demonstrates superior classification of the normal group, correctly identifying 67.67% of cases, compared to 61.38% for XGBoost and 50.54% for MLP.

Multi-scale features improve performance in tree-based models (i.e., XGBoost and CatBoost), KNN and MLP, while other models show no significant changes. Overall, CatBoost emerges as the best model in this study, consistently outperforming others across evaluation metrics and maintaining a better balance across classes as reflected in its higher MCC value.

## 4. Discussion

### 4.1. Towards More Effective SpO_2_-Based Feature Extraction

In this study, we propose a multi-scale approach to enhance feature extraction from SpO_2_ signals. By applying coarse-graining at various timescales, we extend the analysis beyond the original 1 s resolution to larger timescales ranging from 2 to 600 s.

The results reveal three interesting trends in feature relevance: (1) Several features that initially exhibit strong correlations with AHI tend to decline rapidly as the timescale increases. (2) Certain features that are uncorrelated at the original timescale become more strongly correlated after coarse-graining the SpO_2_ signals. (3) Some features undergo correlation polarity shifts, transitioning from positive to negative correlations or vice versa.

Features such as central tendency measure (CTM) [14,39], Lempel–Zip (LZ) complexity [14,39], accumulative time such that SpO_2_ level stays below 95% [14], and kurtosis [20,40] are frequently utilized to assess signal dynamics. These features are approved due to their significance in apnea classification and severity assessment. However, most SpO_2_ biomarkers—except for CTM—lose their relevance with AHI when derived from low-resolution SpO_2_ signals. Notably, their correlation coefficients, which are initially at ρ ≥ 0.5, drop to around 0.3 at a timescale of 30 s. Given that consumer wearable devices store data at resolutions below 60 s, these traditional markers are not suitable for such devices.

The oxygen desaturation index (ODI) has been highlighted as an important feature across studies over a decade [14,23,40,41] for detecting sleep apnea in epoch-wise, subject-wise, and real-time contexts. A potential issue arises in the definition of ODI. ODI refers to the average number of desaturation episodes occurring per hour, where desaturation episodes are defined as a decrease in mean oxygen saturation of ≥3% (over the last 120 s) lasting for at least 10 s [42]. This minimum duration of 10 s implies that this feature cannot be computed with data at lower resolutions. At a 1 s resolution, ODI exhibits a strong correlation with the apnea indicator, with a Spearman’s correlation coefficient of 0.75. However, at a 60 s resolution, the correlation weakens, with the coefficient dropping to 0.25. This decline in correlation strength is evident in Table 2, and the top right scatter plots in Figure 4 visually illustrate this trend.

Interestingly, five features achieve their highest correlation at a timescale of approximately 10 s, with an average difference of 0.216 compared to the original timescale and the largest difference of 0.405 for ZC. These features capture relevant patterns more effectively at a coarser granularity, where transient fluctuations are smoothed, allowing stronger relationships with sleep apnea severity to emerge.

This highlights a key benefit of the coarse-graining technique applied in multi-scale feature extraction, which serves as a low-pass averaging filter for potential noise and disturbances, enhancing feature robustness and revealing underlying patterns more effectively. At the original timescale, the SpO_2_ signal is highly sensitive to external influences, leading to fluctuations that do not necessarily reflect oxygen deficiency but rather noise from movement or other artifacts. The larger timescales reduce the impact of high-frequency noise, enabling the underlying oscillation frequency of the SpO_2_ signal—associated with apnea events—to become more evident.

The above findings generate insights into how different timescales affect the predictive power of features and thus answer RQ1.

Another important finding in this study is that the nine features exhibit high correlation with AHI and maintain a moderate relationship (ρ ≥ 0.5) even at the 60 s timescale. These include two statistical features (central tendency measure and average magnitude of change of SpO_2_) and seven non-linear features (Fuzzy entropy, increment entropy, Kolmogorov entropy, permutation entropy, sample entropy, Shannon entropy, and complex entropy). Notably, non-linear features, such as complex entropy, maintain a strong correlation with the AHI even at timescales as long as 600 s, making them well suited for low-resolution data environments like wearable device applications. This finding is consistent with published studies that reported non-linear features as important sleep apnea indicators. These include entropy measures like approximate entropy (ApEn) [14,39], sample entropy [20], attention entropy [24], and spectral entropy [23], which capture signal complexity and variability. This finding helps us answer RQ2.

Previous studies have mainly investigated features at the 1 s timescale [40,43,44]. A common assumption in signal processing is that a higher sampling rate leads to better signal and feature quality. However, this hypothesis has not been specifically validated in the context of using SpO_2_ for sleep apnea detection. Our results, as shown in Table 2, indicate that this assumption is not entirely accurate and has interesting exceptions, suggesting that sensor researchers should not solely prioritize maximizing the sampling rate but instead explore different rates to balance signal quality with power efficiency. Specifically, our findings highlight the usefulness of non-linear features, demonstrating that even without very high sampling rates, meaningful features can still be extracted for machine learning applications.

### 4.2. A Closer Understanding of Model Performance

Regarding sleep apnea classification, the models struggle the most with the AHI threshold of 5, as this cut-off results in a highly imbalanced dataset. The impact of multi-scale feature engineering is not consistent across all cases and gradually decreases in effectiveness at higher AHI cut-offs (5, 15, and 30). However, it does not negatively affect model performance in any scenario. In addition, at cut-offs 15 and 30, model performance is already relatively strong. Cut-off 15 presents a well-balanced class distribution, while cut-off 30 involves severe cases that are more easily distinguishable.

Overall, multi-scale features enhance evaluation metrics compared to the baseline model at the 1 s timescale. The multi-scale method efficiently impacts the Bayes model in two classification tasks with cut-offs of 5 events/h and 15 events/h. This improvement may stem from the richer data representation provided by multi-scale features, which capture broader temporal patterns and reduce sensitivity to noise or minor variations in the signals. However, despite this improvement, Bayesian performance remains weak. Overall, among the seven models surveyed, CatBoost demonstrates the best performance, effectively classifying classes with minimal bias. This finding addresses the final research question.

### 4.3. Comparison with Previous Study

Many previous studies have aimed to develop computational models for sleep apnea screening using machine learning techniques and have shown promising results. However, these studies vary in terms of the AHI cut-offs used, the number of categories considered (i.e., binary or multiclass classification), the dataset sizes employed for model training and testing, and the metrics used for model performance evaluation, which makes direct comparisons across studies challenging.

We identify several studies that allow direct comparison with part of our results. Three studies report high performance at the cut-off of 5 events/h, but their sample sizes are limited to fewer than 250 sleep records [22,44,45]. Another study using a small proprietary dataset with 320 sleep records [23] achieved the best performance with AdaBoost-Linear discriminant analysis (AB-LDA), which used statistical, spectral, non-linear and clinical-related features derived from SpO_2_. However, their model faced potential overfitting, as evidenced by very high accuracy (78.7–92.9%) and sensitivity (88.9–96.6%) but much lower specificity (50.0–73.5%). A similar issue was observed in the study that used the largest dataset so far (*n* = 8762), which achieved an accuracy of 89.2%, sensitivity of 93.8% and specificity of 56.3% at cut-off of 5 [40]. Their least square boost model performed better for the cut-offs of 15 and 30, achieving accuracy > 85%, sensitivity > 82%, and specificity > 84%. Another recent study used multi-scale attention entropy and trained models with a large dataset (*n* = 5786) but only reported results at AHI cut-offs of 5 and 30 [24]. At cut-off 5, the support vector machine (SVM) had the best performance with accuracy of 74.16%, specificity of 71.08%, sensitivity of 74.84%, and AUC of 0.73. At cut-off 30, the model performed better with an accuracy of 82.57%, specificity of 82.69%, sensitivity of 82.00%, and AUC of 0.82. This study highlighted the importance of non-linear features at multiple scales, but it only focused on one specific feature: the attention entropy.

Our multi-scale feature engineering concept is similar to convolutional neural networks (CNNs), where the data size shrinks across layers, enabling the extraction of generalizable features [46,47]. However, the timescale in those studies is limited to a few seconds and no longer than 60 s. Another similar approach that leverages the finer components of SpO_2_ is the Wavelet transform. In a previous study, SpO_2_ signals were decomposed into six sub-bands [48], and Shannon entropy features were extracted from each sub-band to train the model, achieving an AUC of 0.96 for epoch-wise classification. However, these studies did not focus on subject-wise classification, making direct comparison with this study unfeasible. In addition to improving model performance, our approach offers the advantages of being less computationally expensive and providing better interpretability.

## 5. Conclusions

In this study, we proposed a multi-scale feature engineering approach for developing machine learning models for sleep apnea detection and severity classification. We conducted a comprehensive evaluation of its impact on feature relevance and model performance.

A key finding is that while SpO_2_ signals exhibit reduced resolution when coarsely grained at larger timescales, some features extracted at these timescales show stronger correlations with the AHI. This result is important, as it suggests that sleep apnea severity could potentially be classified using lower-resolution data, such as those obtained from consumer-grade wearable devices (e.g., Fitbit, Apple Watch, and Google Pixel Watch). These devices, while more limited in capturing complex and high sampling rate signals like EEG or ECG compared to the gold-standard polysomnography, may still yield relevant insights for sleep apnea detection in daily life. Furthermore, our analysis reveals that multi-scale features help alleviate model bias, resulting in more balanced performance across all classes.

The current study has two primary limitations. First, the models were trained and tested using only one dataset. Future efforts should validate these with additional datasets encompassing more diverse populations. Second, while model bias was alleviated through multi-scale feature engineering, performance was still affected by dataset imbalance. As a next step, we plan to apply techniques to mitigate the effects of this imbalance, such as adjusting class weights and decision thresholds.

## Figures and Tables

**Table 1:** Demographic information for the subjects whose data are used for model development in this study.

	Categories	SHHS1
Number of subject ^1^		4664
Age		65.88 ± 11.01
BMI		28.24 ± 5.04
Gender	Female	2287 (49.04%)
	Male	2377 (50.96%)
AHI		18.66 ± 15.4
Binary (AHI cut-off = 5)	Normal	634 (13.59%)
	Patient/have apnea	4030 (86.40%)
Binary (AHI cut-off = 15)	Normal & mild	2426 (52.02%)
	Moderate & severe	2238 (47.98%)
Binary (AHI cut-off = 30)	Non-severe	3 840 (82.33%)
	Severe	824 (17.67%)
Multiclass	Normal	634 (13.59%)
	Mild	1792 (38.42%)
	Moderate	1414 (30.32%)
(AHI cut-offs: 5, 15, 30)	Severe	824 (17.67%)

^1^ After pre-processing steps.

**Table 2:** The table presents features that exhibit notable trends across multiple timescales. It includes the highest and lowest correlation values observed, the median correlation value, and the specific timescale at which the highest correlation occurred.

Trend	Features	Description	Correlation Coefficient Score (ρ)			Timescale of Highest ρ
			Highest	Lowest	Median	
	M	Percentage of the signal at least x% below median SpO_2_, by default x = 2	0.54	0.18	0.40	2 s
	ODI	The oxygen desaturation index	0.77	0.2	0.48	3 s
	CA	Integral of SpO_2_ below the x SpO_2_ level normalized by the total recording time, by default x = AV, here 93	0.54	0.16	0.4	1 s
	POD	Time of oxygen desaturation event, normalized by the total recording time	0.75	0.20	0.41	1 s
Showing decreasing trend	AODmax	The area under the oxygen desaturation event curve, using the maximum SpO_2_ value as baseline and normalized by the total recording time	0.68	0.20	0.40	1 s
	AOD100	Cumulative area of desaturations under the 100% SpO_2_ level as baseline and normalized by the total recording time	0.71	0.20	0.42	1 s
	ApEn	Approximate entropy with, by default, m = 1, r = 0.25 times the standard deviation of the data	0.85	0.11	0.72	5 s
	alpha	Long-term correlations for non-stationary process, using non-overlapping windows	−0.57	−0.08	−0.44	5 s
	alphaOverlap	Long-term correlations for non-stationary process, using overlapping windows	−0.57	−0.12	−0.43	5 s
	DistEn	Distribution entropy	0.52	0.23	0.43	1 s
Reach	ZC	Mean crossing	0.61	−0.01	0.45	20 s
stronger	LZ	Lempel–Zip complexity	0.56	0.13	0.43	10 s
correlation at higher	PRSAc	Phase-rectified signal averaging capacity. With d the fragment duration, here d = 10	−0.68	0.03	−0.17	6 s
timescale	CondEn	Corrected conditional entropy	0.53	0.24	0.42	10 s
	DispEn	Dispersion entropy	0.57	0.24	0.45	15 s
Show	hjm	Hjorth mobility	0.45	−0.21	0.22	20 s
correlation	hjc	Hjorth complexity	−0.46	0.10	−0.27	7 s
polarity	PhasEn	Phase entropy	−0.60	0.07	0.18	3 s
shifts	ComplexEn	Complex entropy	−0.86	0.31	−0.76	10 s
	CTM	Central tendency measure with radius ρ, by default ρ = 0.25	−0.82	−0.37	−0.75	15 s
	Mean abs change	Average magnitude of change between consecutive SpO_2_ values	0.84	0.28	0.71	15 s
Constant high	FuzzEn	Fuzzy Entropy	0.86	0.38	0.76	15 s
high	IncrEn	Increment entropy	0.86	0.39	0.77	15 s
relationship	K2En	Kolmogorov (K2) entropy	0.81	0.37	0.73	9 s
	PermEn	Permutation entropy	0.86	0.37	0.76	3 s
	SampEn	Sample entropy	0.80	0.36	0.71	5 s
	ShannonEn	Shannon entropy	0.66	0.28	0.55	4 s
	ComplexEn	Complex entropy	−0.86	0.31	−0.76	10 s

**Table 3:** Classification results for each models at each task with 3 settings.

	Acc (%)		Pre (%)		Sen (%)		F1-Score		MCC		AUC	
	**Multi-Scale**	**Baseline**	**Multi-Scale**	**Baseline**	**Multi-Scale**	**Baseline**	**Multi-Scale**	**Baseline**	**Multi-Scale**	**Baseline**	**Multi-Scale**	**Baseline**
Cut_off_5												
Bayes	73.59	67.17	96.77	96.75	71.84	64.16	0.82	0.77	0.41	0.35	0.83	0.84
DT	85.38	84.35	91.91	91.67	91.10	90.07	0.92	0.91	0.39	0.37	0.65	0.69
KNN	87.50	87.11	88.21	87.68	98.73	98.99	0.93	0.93	0.29	0.24	0.87	0.85
LG	88.54	88.94	93.62	91.33	93.08	96.35	0.93	0.94	0.52	0.46	0.92	0.91
XGB	88.66	88.37	92.32	91.99	94.76	94.80	0.94	0.93	0.48	0.47	0.91	0.91
CatBoost	89.63	89.10	92.27	91.44	96.06	96.42	0.94	0.94	0.51	0.47	0.92	0.92
MLP	87.13	86.24	92.01	91.66	93.20	92.49	0.93	0.92	0.43	0.40	0.89	0.87
Cut_off_15												
Bayes	79.58	77.17	84.12	87.66	70.92	61.08	0.77	0.72	0.60	0.56	0.86	0.86
DT	81.36	80.80	81.62	80.96	79.01	78.52	0.80	0.80	0.63	0.62	0.79	0.81
KNN	83.57	82.20	85.06	84.26	79.84	77.42	0.82	0.81	0.67	0.64	0.91	0.90
LG	87.05	86.49	87.69	87.24	85.01	84.20	0.86	0.86	0.74	0.73	0.94	0.94
XGB	86.11	86.08	86.31	86.56	84.49	84.09	0.85	0.85	0.72	0.72	0.94	0.94
CatBoost	87.17	86.82	87.59	87.43	85.42	84.79	0.87	0.86	0.74	0.74	0.95	0.94
MLP	84.89	82.85	84.47	82.72	84.00	81.33	0.84	0.82	0.70	0.66	0.93	0.91
Cut_off_30												
Bayes	85.92	88.05	57.41	64.57	79.53	72.21	0.67	0.68	0.59	0.61	0.89	0.91
DT	90.53	89.47	74.38	71.45	71.10	67.67	0.73	0.69	0.67	0.63	0.79	0.83
KNN	90.74	91.07	87.94	83.85	55.20	61.47	0.68	0.71	0.65	0.67	0.94	0.94
LG	92.02	92.79	74.51	84.15	83.66	73.08	0.79	0.78	0.74	0.74	0.96	0.96
XGB	92.86	92.12	82.27	80.39	76.17	73.54	0.79	0.77	0.75	0.72	0.96	0.95
CatBoost	93.47	92.67	84.37	82.55	77.50	74.34	0.81	0.78	0.77	0.74	0.96	0.96
MLP	91.92	90.85	77.83	74.64	76.16	73.42	0.77	0.74	0.72	0.69	0.95	0.94
Multiclass												
Bayes	57.70	57.36	57.66	57.87	58.25	55.57	0.58	0.56	0.41	0.40		
DT	60.85	58.67	60.87	58.24	60.02	57.55	0.60	0.58	0.45	0.42		
KNN	62.32	60.91	66.13	63.07	55.05	53.92	0.57	0.55	0.46	0.44		
LG	67.54	68.37	67.43	69.32	65.09	64.49	0.66	0.66	0.54	0.55		
XGB	69.28	68.29	69.86	68.84	67.16	65.60	0.68	0.67	0.56	0.55		
CatBoost	70.46	68.54	71.88	69.85	67.64	64.75	0.69	0.66	0.58	0.55		
MLP	62.72	60.14	62.40	59.49	61.49	58.78	0.62	0.59	0.47	0.44		

## Data Availability

The SHHS dataset used in this study are available at https://sleepdata.org/datasets with the permission of NSRR. The original contributions presented in this study are included in the article. Further inquiries can be directed to the corresponding author.
